# Sesame Detection in Food Using DNA-Functionalized Gold Nanoparticles: A Sensitive, Rapid, and Cost-Effective Colorimetric Approach

**DOI:** 10.3390/bios14080377

**Published:** 2024-08-03

**Authors:** Pablo Llano-Suárez, Adrián Sánchez-Visedo, Inmaculada Ortiz-Gómez, María Teresa Fernández-Argüelles, Marta Prado, José Manuel Costa-Fernández, Ana Soldado

**Affiliations:** 1 Department of Physical and Analytical Chemistry, University of Oviedo, c/Julián Clavería, 8, 33006 Oviedo, Spainadrian.visedo@inl.int (A.S.-V.); fernandezteresa@uniovi.es (M.T.F.-A.); jcostafe@uniovi.es (J.M.C.-F.); 2 International Iberian Nanotechnology Laboratory, Av. Mestre José Veiga Sthis n, 4715-330 Braga, Portugal; marta.prado@usc.es

**Keywords:** food safety, AuNPs, MNAzymes, analytical signal amplification, color analysis, sesame allergen

## Abstract

Food safety control is a key issue in the food and agriculture industries. For such purposes, developing miniaturized analytical methods is critical for enabling the rapid and sensitive detection of food supplements, allergens, and pollutants. Here, a novel bioanalytical methodology based on DNA-functionalized gold nanoparticles (AuNPs) and colorimetric detection was developed to detect the presence of sesame (a major allergen) through sesame seed DNA as a target, in food samples. The presence of sesame DNA induces controlled nanoparticle aggregation/desegregation, resulting in a color change (from blue to red) proportional to sesame DNA concentration. The incorporation of multicomponent nucleic acid enzymes (MNAzymes) in this strategy has been carried out to perform an isothermal signal amplification strategy to improve the sensitivity of detection. Also, open-source software for color analysis was used to ensure an unbiased visual color-change detection, enhancing detection accuracy and sensitivity and opening the possibility of performing a simple and decentralized analyte detection. The method successfully detected the presence of sesame DNA in sesame seed, sesame oil, olive oil, and sunflower oil. In brief, the developed approach constitutes a simple and affordable alternative to perform a highly sensitive detection of DNA in food without complex methodologies or the requirement of expensive instrumentation.

## 1. Introduction

Sesame, *Sesamum indicum*, is a seed used in many dishes, particularly in Middle Eastern cuisine, in different ways such as in paste (tahini), a sweet (halva), a topping (like in hamburger buns), or a cooking oil [1], among others. Furthermore, sesame is also a common food allergen, and reports of sesame allergies have been increasing worldwide [2]. Sesame can induce adverse reactions in 0.1% to 0.2% of the general population. Some seeds, particularly their oils, may cause delayed hypersensitivity (T-cell-mediated), often manifesting as allergic contact dermatitis. Although ingestion is the primary mode of exposure, inhalation and skin contact, especially in occupational settings, can also trigger reactions. Immunoglobulin E (IgE)-mediated sesame food allergy typically presents with clinical symptoms early in life (around 2 years of age) and may be persistent. This allergy can cause a range of adverse effects, including mucocutaneous reactions such as atopic dermatitis and urticaria, gastrointestinal symptoms, respiratory issues, and life-threatening anaphylaxis. Sesame allergies have been connected with at least five different proteins, with Ses i 1, Ses i 2, and Ses i 8 being the most representative. Ses i 1 is a 2S albumin with a low concentration of sulfur and 40% homology to Brazilian nut, sunflower seed, and castor bean allergens. Ses i 2 and Ses i 8 is a 2S and 8S albumin with rich sulfur.

Currently, there are several methods for sesame detection including enzyme-linked immunoassay (ELISA) [3], polymerase chain reaction (PCR) [4,5,6], and targeted proteomics based on liquid chromatography coupled with mass spectrometry (LC-MS) [7,8]. Although these methods are highly sensitive and selective, most of them require complicated sample pretreatment, which makes on-site field measurement difficult or impossible [9,10], which is primarily needed for allergen determination in food. Additionally, the conventional method application typically requires specialized and expensive laboratory equipment, as well as trained personnel to interpret the results [11]. Today, there is a need to develop new analytical methodologies that allow for the on-site and simple screening of allergens in food using simple instrumentation and small volumes of samples and reagents. Furthermore, the use of biosensors in chemical analysis offers significant cost benefits. As mentioned earlier, biosensors require fewer reagents and specialized equipment, thereby reducing costs. In addition, biosensors enable faster and simpler analyses, reducing both labor costs and analysis time. In contrast, conventional methods such as PCR, while highly accurate and sensitive, are more expensive due to the need for expensive reagents, equipment, and specialized technical personnel, which significantly increases the overall cost of the analysis [12,13,14]. The detection of sesame seed DNA targets in food could allow for an accurate identification of its presence in food products, and quantitative PCR is required. Several methods have been developed for the detection of sesame DNA in food products, many of them based on the PCR technique [15]. Alternatively, a combination of inorganic nanoparticles with MNAzymes for the amplified detection of nucleic acids appears as a powerful strategy for developing simple and highly sensitive bioassays for DNA detection [5,16].

Gold nanoparticles have unique optoelectronic properties that make them ideal for biosensor development. Also, AuNPs can be easily functionalized with different biomolecules increasing their applicability in the development of optical biosensors. These biomolecules act as recognition elements, binding selectively to target analytes such as specific proteins or nucleic acids, among others. This interaction induces changes in the nanoparticle’s optical properties, including shifts in their surface plasmon resonance, which can be detected optically. The ability to precisely control the bioconjugation of gold nanoparticles with DNA enhances the specificity and sensitivity of biosensors, enabling them to detect and quantify target molecules with high accuracy and efficiency. Thus, the combination of gold nanoparticles’ optoelectronic properties with bioconjugation techniques forms the foundation for advanced biosensing technologies with diverse applications in clinical diagnostics [12]. Also, their size-dependent optical properties, in particular their surface plasmon resonance, enable sensitive detection mechanisms. The interaction of light with these nanoparticles can cause them to change color, providing a visual indication of molecular interactions or changes in their environment. This phenomenon is due to the ability of gold nanoparticles to exhibit plasmonic effects, where the collective oscillation of free electrons on their surface results in enhanced light absorption and scattering. The optoelectronic properties of gold nanoparticles offer the possibility of observing a change in color as a result of changes in the distance between them, making them invaluable in the development of highly sensitive biosensors capable of detecting minute molecular interactions with precision and reliability [4].

A different DNA detection technique that has a lot of potential for fast and easy assays is the use of two gold nanoparticle sets bioconjugated with different DNA probes which recognize the target sequence inducing aggregation of AuNP sets and color changes due to the interparticle plasmon resonance from red (dispersed AuNPs) to purple or blue (aggregated AuNPs) [17,18,19]. The simplest way to do so is the use of gold nanoparticles that have been bioconjugated to DNA/RNA chains that are each complementary to half of the target analyte, so in their presence, they come together and aggregate [20]. This results in a change in color, from red to blue, and can be detected visually. However, this simple method is usually not sensitive enough to achieve limits of detection low enough for many DNAs, needing a previous amplification step. One such way is the use of MNAzymes, a synthetic DNA chain that acts as an enzyme [21].

MNAzymes, a class of nucleic acid enzymes have emerged as powerful tools [18,20] for the optical detection of genetic biomarkers. These enzymes are composed of single-stranded sequences of either DNA or RNA organized into a catalytic core, sensor arms that are complementary to the target sequence, and substrate-binding arms complementary to another sequence known as the DNA linker [22]. The linker contains two DNA bases where cleavage occurs. This cleavage only happens in the presence of a target, upon hybridization with the sensor arms of the MNAzyme. In contrast, in the absence of the target, the linker sequence remains intact. However, in the presence of the target, the linker is cleaved into two shorter strands. Thus, the catalytic activity of MNAzymes can be modulated by the presence of genetic biomarkers, making them ideal candidates for developing highly specific bioanalytical strategies [23]. Specifically, when coupled with gold nanoparticles (AuNPs) as detection tags, MNAzymes can be used for the direct detection of nucleic acids in a protein, isothermal, and optical format allowing for signal amplification [24]. MNAzyme amplification makes use of a linker single-stranded DNA chain that is complementary to two nucleotide chains that are bioconjugated to the gold nanoparticles. The presence of the linker produces aggregation of the gold nanoparticles, and it is cleaved when the MNAzymes become active if the DNA target is present in the solution [25]. The use of MNAzymes presents several advantages, and signal amplification increases from the probe molecule hybridized to the target nucleic acid sequence. Also, the advantages of MNAZymes amplification include specific detection, a wide dynamic range, ease of use, and reproducibility.

Following this procedure, in this work, we have established a novel colorimetric method based on bioconjugated gold nanoparticles, combining MNAzyme amplification and free color image software-assisted tools to achieve low detection limits for the determination of sesame. The image software tool has been utilized for chemical analysis based on color analysis, offering a powerful, fast, and cost-effective method to detect target analytes based on the color changes in digital images captured by cameras [26,27]. A digital camera has been used as an optical sensing element to acquire chemical information through the image processing technology of a TLC plate with different sample spots [28,29]. Image color analysis significantly enhances the sensitivity of colorimetric assays in chemical analysis by eliminating the inherent subjectivity of human visual perception [30]. By using digital cameras to capture images of samples on the TLC plate, a precise digital representation of the colors is obtained. These photographs are processed with advanced software, allowing for the accurate and semiquantitative detection of color changes that are difficult to discern with the naked eye [31]. This method not only improves the sensitivity of the assay but also ensures more reproducible and reliable results, regardless of the observer’s subjectivity and varying ambient light conditions. Consequently, the proposed approach enables a sensitive, rapid, robust, low-cost, and user-friendly method for detecting sesame in food products. The colorimetric sensor developed here demonstrates good sensitivity, specificity, precision, and accuracy for detecting sesame as an allergen.

## 2. Materials and Methods

### 2.1. Reagents and Materials

All experiments were carried out with analytical-grade chemical reagents and all aqueous solutions were made using reverse-osmosis type quality water, with a resistivity of 18.2 MΩ·cm (Millipore, Bedford, MA, USA). Agarose was obtained from Bio-Rad (Biorad, Hercules, CA, USA). Hydrogen tetrachloroaurate trihydrate (HAuCl_4_∙3H_2_O) (CAS 16961-25-4), sodium citrate tribasic dihydrate (Na_3_C_6_H_5_O_7_∙2H_2_O) (CAS 6132-04-3), Magnesium Chloride Hexahydrate (MgCl_2_∙6H_2_O) (CAS 7791-18-6), Trizma^®^ Hydrochloride (NH_2_C(CH_2_OH)_3_∙HCl) (CAS 1185-53-1), Potassium Chloride (KCl) (CAS 7447-40-7), and Tween-20 (CAS 9005-64-5) were purchased from Sigma-Aldrich (St. Louis, MO, USA; (Sigma Aldrich, Darmstadt, Germany). Thiolated polyethylene glycol (mPEG-SH1000) (CAS 345260-48-2) was obtained from Laysan Bio, Inc. (Huntsville, AL, USA; (Laysanbio, Alabama, AL, USA). Thin Layer Chromatography (TLC) Aluminum Sheets Silica gel 60 F254 and Analtech TLC Uniplates™, where the C18-silica gel matrix is immobilized onto a glass support, were purchased from Merck KGaA (Merck, Darmstadt, Germany). The strands used to functionalize the gold nanoparticles (AuNPs) and the evaluated sesame DNA sequences (AF_Ses 1 to 4) were purchased from BioBasic (Markham, Ontario, Canada), and their corresponding MNAzymes are listed in Table 1. Additionally, the melting temperatures (Tm) of the DNA sequences used in this work are summarized in Table 1. The linker strand was obtained from Integrated DNA Technologies (IDT, Coralville, LA, USA). According to our previous research work [18], the optimal hybridization of DNA strands in the amplification assay using MNAzymes is achieved when sequences exhibit a melting temperature near 50 °C. Among the sequences evaluated in this study, Ses3 demonstrated a melting temperature of 48.9 °C, highlighting its suitability as an efficient recognition probe in the amplification phase employing MNAzymes. Thus, AF_Ses3 was used in the development of this work.

### 2.2. Instruments and Software

Spectroscopic measurements were carried out using a Thermo GENESYSTM 10S UV–visible spectrophotometer (Thermo Scientific, Waltham, MA, USA). The size, shape, and distribution of the AuNPs were carried out by transmission electron microscopy (TEM) JEM 2000EXII de JEOL. In addition, the bioconjugated and non-bioconjugated AuNPs’ size and the polydispersity index (PDI) were analyzed by DLS Zetasizer Nano S from Malvern. All pH measurements were carried out on a Mettler ToledoTM pH meter. The PowerPac 300 from Biorad was used for the electrophoresis gels. The TLC plates were photographed using a Cannon K-X (Pentax Corporation, Tokyo, Japan) digital camera and appropriate settings to maintain color integrity and white balance. These photographs were treated using Ilastik, an open-source software that allows for the classification of complex textures by different properties, such as their color [31].

### 2.3. Selection of the MNAzyme Sequences

The maturase K (MatK) gene was selected for the design of the MNAzyme sequences. The MatK gene is a chloroplast gene that has been suggested to be a “barcode” for terrestrial plants since it is considered one of the most variable coding genes of angiosperms [22]; therefore, it is possible to identify regions of this gene that can provide a high level of specificity for the design of analytical methods. Sequences corresponding to the MatK gene from *Sesamum indicum* were retrieved from GeneBank (accession numbers: JN637766, NC016433, and AJ429340) and were aligned, and a consensus sequence obtained with Geneious Prime^®^ and was used for the design of the qPCR method using the software Primer Express 3.0 (Applied Biosystems, Foster City, CA, USA). From all the primers and probes suggested by the Primer Express software V3.0.1, different combinations were tested in silico with Geneious software V3.0.1 aligned with both sesame DNA sequences and DNA sequences from other seeds and spices frequently used in food products, namely sunflower, pine nut, peanut, rice, wheat, maize, chickpea, mustard, and different varieties of pepper and flax seed (accession numbers: AB084496, AB354272, AY215805, HM544115, EU307346, AY964640, EU434287, AB198874, AB198875, AB198876, EF537299, JF276754, EF537293, EF537275, EF537289, EF537305, and EF537271), for the best results, and to avoid interferences from other species.

Additionally, BLAST analysis was also used to identify regions of local similarity between the chosen nucleotide and homolog sequences. Sequence homology with *Sesame indicum* sequences was confirmed, while mismatches with primer sequences were also confirmed with tested seeds and spices. Once designed and tested, selected primers were used for the design of the different MNAzyme sequences considering the areas of interest on the MatK gene region.

**Figure 1:**
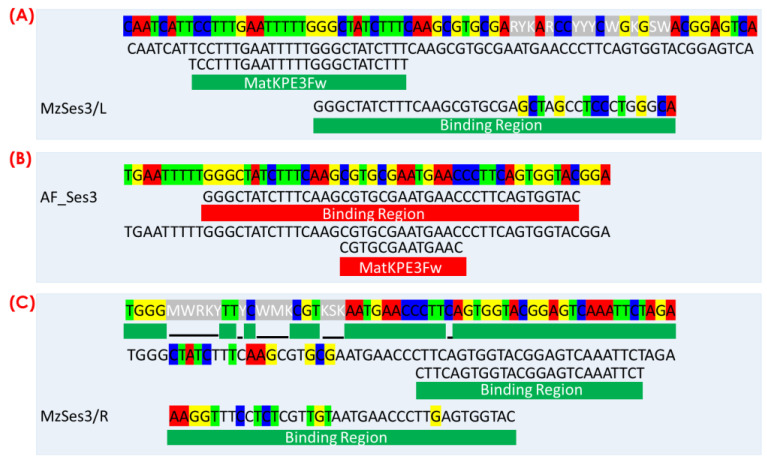
(**A**) Alignments of the consensus sequence from the maturase of *Sesamum indicum* with MatKPE3Fw and MzSes3/L showing the complementarity region with the MatK gene and the non-complementary region designed to hybridize with the sequence attached to the gold nanoparticle, (**B**) with matKPE3Probe and AF_Ses3 showing the complementarity region with the MatK gene and the specific region recognized by the MNZyme, (**C**) with matKPE3Rev and MzSes3/R showing the complementarity region with the MatK gene and the non-complementary region designed to hybridize with the sequence attached to the gold nanoparticle.

Therefore, from the selected region, several combinations were selected to include both the specific regions and a DNA sequence, intended to hybridize with the complementary sequence attached to the nanoparticle. Likewise, a nucleotide sequence was designed to hybridize with an internal complementary sequence recognized by the MNAZyme. Figure 1A–C show the detail of the alignment of the consensus sequence.

### 2.4. Synthesis of AuNPs

Gold nanoparticles were prepared following the Turkevich–Frens reaction [32,33,34]. In brief, 1 mL of hydrogen tetrachloroaurate trihydrate (HAuCl_4_∙3H_2_O) 25 mM was added to a 250 mL Erlenmeyer flask (pre-cleaned using Aqua Regia) filled with 98 mL ultrapure water. The liquid content in the flask was heated to boiling under constant intense mechanical stirring. Then, 1 mL of sodium citrate tribasic dihydrate (Na_3_C_6_H_5_O_7_∙2H_2_O) 33 mg/mL was added, and the system was heated for 10 min until the solution turned deep red. Finally, the reaction was stopped by the introduction of the flask into an ice bath. Dynamic light scattering (DLS) was employed to establish the size dispersion of the obtained AuNPs. Also, the size and morphology of synthesized AuNPs were studied using transmission electron microscopy (TEM) measurements.

To maintain the AuNPs’ dispersion and prevent aggregate formation, the citrate-stabilized nanoparticles were stored in 0.01% of Tween-20 solution. Also, the synthesized gold nanoparticles were preconcentrated to 100 nM by adding ultrapure water containing 0.01% Tween-20, and the solution was stored at 4 °C until use. To calculate the molar concentration of gold nanoparticles [18], we first determined the particle size using transmission electron microscopy, and also, we measured their maximum absorbance at 521 nm. Using these data, the volume of the AuNPs was calculated to be 1.15 × 10^3^ nm^3^, and the mass density of atomic gold was calculated to be 1.939 × 10^−20^ mol/nm^3^. These values were based on an extinction coefficient of 2.33 × 10⁸ M^−1^ cm^−1^ at λ = 521 nm for 15 nm AuNPs. Additionally, the calculated particle mass was 2.23 × 10^−17^ g, resulting in a molar mass of AuNPs of 1.34 × 10^−7^ g/mol.

### 2.5. AuNP Bioconjugation

The synthesized AuNPs were bioconjugated employing two thiol-modified DNA sequences (DNA Strand 1, named Agg1, and DNA Strand 2, named Agg4), complementary each to half of the linker strand. The two sets were prepared by mixing 100 μL of AuNPs 100 nM with 15 μL of DNA 100 µM into an Eppendorf tube, adding 145 μL of ultrapure water, and 40 μL of Tween-20 0.1%. After 5 min of reaction, 100 μL of Trisodium Citrate HCl buffer 90 mM at pH 3 was added to the mixture. Then this mixture was incubated at an isothermal temperature (room temperature) for 30 min. The AuNP’s surface was then blocked from further interactions using thiolated polyethylene glycol (mPEG-SH1000). Thus, 200 μL of mPEG-SH1000 was added to the AuNP solution, which was then heated to 60 °C for 30 min. The particles were purified via 3× centrifugation at 8000× *g* for 30 min and, each time, discarding the supernatant and resuspending the nanoparticles using 0.01% Tween-20. The final product was diluted to 900 μL, obtaining a final concentration of 11 nM (determined based on the extinction coefficient of 2.33 × 108 M^−1^·cm^−1^ at λ = 521 nm) [18].

### 2.6. Standard Measurement Protocol

In a typical detection assay, 1 μL of MNAzyme buffer (0.1 M Tris-HCl, 0.5 M KCl, pH = 8.3), 1 μL of MgCl_2_ 300 mM, 1 μL of MNAzyme solution (4 μM in both MNAzyme strands), 1 μL of 1 µM linker, and 6 μL of the test sample were added to an Eppendorf tube, which was heated to 50 °C for 1h to activate the MNAzymes. Then, 5 μL of both DNA-AuNP was added to the solution and incubated at 50 °C for 20 min to allow the linker to hybridize. In the presence of the DNA target (AF_Ses3), see Figure 1B, a catalytic procedure cleaves the DNA linker in two shorter sequences and avoids the AuNP aggregation. Here, concretely, the AF_Ses3 sequence was used as a target in the amplification assay using MNAzmes. This sesame DNA sequence exhibits a melting temperature near 50 °C. Among the sequences evaluated in this study, Ses3 demonstrates a melting temperature of 48.9 °C, highlighting its suitability as an efficient recognition probe in the amplification phase employing MNAzymes [18].

For naked eye detection or solid phase detection, 3 µL of each assay was spotted on TLC plates.

### 2.7. Image Capture and Processing

The TLC plates were photographed using a digital camera (Pentax Corporation) and appropriate settings to maintain color integrity and white balance. The setting conditions employed to take photos were ISO 400, shutter speed 1/1600, aperture value f/5, focal distance 35 mm, and resolution 4288 × 2848 [30]. The spots present in the TLC plate were imaged using a cube light box with the camera placed in the front of the box. This box contained two white LEDs with a color temperature of 6500 K and a spectrum ranging from 400 to 700 nm for RGB measurements. The LED emits a cold white light. The interpretation of results from the TLC plates could be influenced by the naked eye, so we used software that can automatically identify positive and negative results based on the visual readout to remove this uncertainty (Ilastik software V1.4.0) [31].

### 2.8. Extracting DNA from Food Samples

For the analysis of real samples, sesame DNA was extracted from sesame oil and sesame seeds using the Promega Wizard DNA extraction kit [32]. Olive and sunflower oil, as well as wheat flour DNA, were extracted to be used as controls. To extract the oils, 40 mL of oil was placed on a 50 mL centrifuge tube with the appropriate lysis buffers. After 10 min under agitation, 3 mL of the precipitation solution was added. This mixture was homogenized and then centrifuged for 20 min at 4000× *g*, producing 3 layers, from the top to the bottom: an organic phase (hexane), an aqueous phase, and a solid phase. The organic phase was removed carefully using plastic pipette tips, and the aqueous phase was recovered to ensure that there was no contamination from the solid phase. The recovered aqueous phase was placed in a 15 mL centrifuge tube, taking note of the obtained volume. After that, following the Promega Wizard DNA extraction kit instructions, 50 μL of magnetic nanoparticles were added to the tube, and then isopropanol was added in a 0.9 ratio of the volume recovered. After 1 h of incubation under agitation, the DNA was bound to the magnetic nanoparticles, so the supernatant could be removed after placing the tube in a magnetic rack. After 3 washes using 2 mL of ethanol solution at 70%, the magnetic nanoparticles were dried in the tube for 20 min and resuspended in 100 μL ultrapure water. This volume was placed on an Eppendorf tube where it was vortexed and heated to 65 °C for 5 min to free the DNA from the magnetic particles. Finally, the Eppendorf was placed on a magnetic rack, and the solution was recovered.

This methodology was adapted to the extraction of DNA from solids (sesame seeds) by placing 20 g of the solid in 40 mL hexane and recovering the hexane after 8 h. The DNA extraction then proceeded analogously to the oil extraction. The detection assay was performed under optimal experimental conditions.

## 3. Results

### 3.1. Principle of Proposed Colorimetric Method

**Scheme 1:**
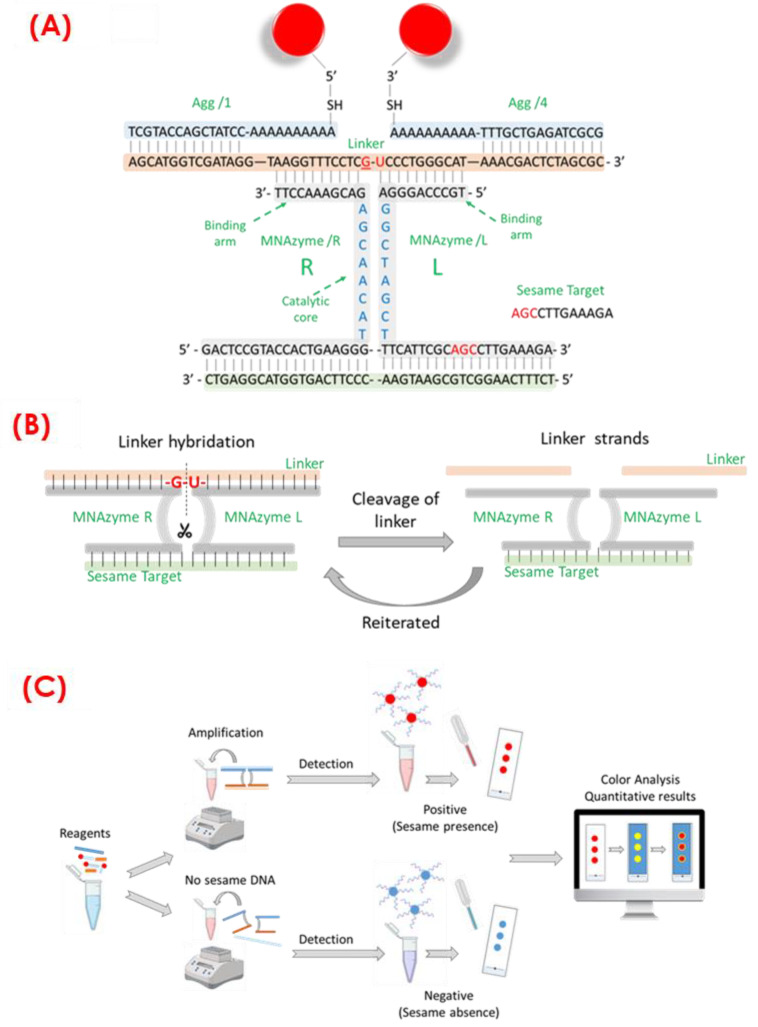
(**A**) A scheme illustration of an MNAzyme design using DNAzymes and (**B**) the full MNAzyme system amplification, showing the cleavage of the linker. The green line represents the sesame DNA target, the gray lines represent the MNAzymes R and L, and the orange lines represent the linker strand. In red are the DNA bases (cleaving point for the MNAzymes), and (**C**) shows the experimental workflow of sesame DNA detection and quantification.

The proposed colorimetric assay for sesame DNA detection, based on AuNPs bioconjugated with the two DNA strands selected (Agg1 and Agg4) and MNAzyme amplification, is illustrated in Scheme 1A. MNAzyme amplification makes use of a “linker” single-stranded DNA chain (Linker in Scheme 1B) that is complementary to half of the two chains that are bioconjugated to the gold nanoparticles and to both arms of the MNAzyme system (see Scheme 1A, MNAzyme/R, and MNAzyme/L).

The MNAzyme amplification process starts with the activation of the catalytic core of the MNAazyme by the target, thus bringing together MNAzymes R and L. Then, the linker may subsequently be recognized by the active MNAzyme complex, which irreversibly cleaves it into two DNA nucleotides. Subsequently, the pieces of the linker dehydrate, allowing for the active MNAzyme complex to sequentially cleave many linkers (amplification). The concentration of the DNA target in the sample determines the quantity of cleaved linkers, and it is associated with the amount of sesame in the sample. Finally, visual quantification of the linkers or the cleavage products is achieved by labeling both ends with DNA-functionalized AuNPs (detection). The higher the concentration of DNA in the sample, the higher the percentage of free AuNPs and the closer the solution’s color is to bare AuNPs (red color). Conversely, if the DNA concentration is lower, AuNP aggregates are more prevalent, and the color of the test shifts to blue values.

The color change of the colloidal dispersion due to the presence of the target can be detected by placing a 3 µL drop onto a TCL plate; as a result, the evolution of the colors while changing the DNA target concentration can be distinguished by the naked eye. Thanks to the strong signal amplification achieved by the use of the described genetic amplification approach, this assay allows for the visual detection of low concentration levels of the targets by the naked eye. In order to enhance the sensitivity of this methodology and avoid errors related to human-eye color interpretation, image processing software (Ilastik) has been employed (see Scheme 1C). In this case, using image processing software aims to improve sensitivity in visual detection.

These color changes can be visualized by the naked eye and by the UV-Vis absorption spectra of AuNPs. There is a displacement of the maximum absorbance wavelength from blue-purple for aggregated AuNPs (approx. 540 nm) to red for dispersed AuNPs (approx. 520 nm), and it can be measured using UV-Vis spectroscopy. The optical properties of AuNPs change depending on the distance between them. A shorter linker single-stranded DNA chain results in a larger absorption wavelength shift of the AuNPs because the distance between them is smaller. In contrast, longer linker single-stranded DNA chains generate greater separation between the nanoparticles, leading to smaller wavelength shifts. In this work, a linker chain of 54 base pairs was used, which, as mentioned above, produced a blue shift of 20 nm.

MNAzyme structures contain a catalytic core flanked by two binding arms (R and L in Scheme 1A). Also, the linker strand contains two DNA bases where cleavage occurs when it is hybridized to the MNAzyme in the presence of the target (Scheme 1B). The catalytic core is responsible for the cleavage of the DNA linker, whereas the binding arms are used to hybridize to the linker through Watson–Crick base pairing [35]. The sequences of the binding arms in these MNAzymes have little effect on the catalytic activity. The cleavage site of the linker is a dinucleotide junction located between the two binding arm domains within the linker. The cleavage mechanism involves attacking the adjacent phosphodiester linkage via the 2′-hydroxil group of the upstream nucleotide within the cleavage junction [35,36]. In the absence of sesame DNA, the MNAzyme arms (R and L) do not undergo hybridization, and the catalytic core does not cleave the DNA linker. As a result, genetic amplification does not take place.

### 3.2. Characterization of Synthesized and Bioconjugated AuNPS

**Figure 2:**
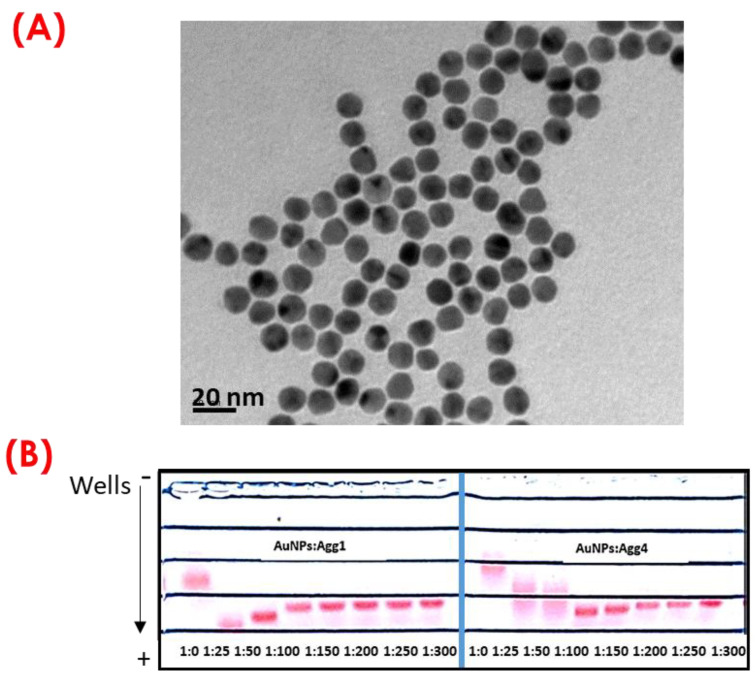
(**A**) TEM images of dispersed AuNPs−DNA with sesame DNA. (**B**) Agarose gel of AuNP bioconjugates formed by incubation at indicated AuNPs:Agg1 (**left**) and AuNPs:Agg4 (**right**) molar ratios of 1:0, 1:25, 1:50; 1:100, 1:150, 1:200, 1:250, and 1:300.

The size of synthesized AuNPs was confirmed using dynamic light scattering (DLS) and transmission electron microscopy (TEM). The size of AuNPs was found to be 13.6 ± 1.6 nm using DLS analysis and 15.2 ± 1.2 nm using TEM, see Figure 2A. After synthesizing AuNPs, these were bioconjugated with Agg1 and Agg4 DNA sequences using the procedure described in Section 2.5. AuNP bioconjugation. Different molar ratios of AuNPs: Agg1 and AuNPs: Agg4 were tested in the bioconjugation reaction to optimize the AuNP bioconjugates: 1:0, 1:25, 1:50, 1:100, 1:150, 1:200, and 1:300. All the AuNPs bioconjugated were analyzed via gel electrophoresis, using agarose gels at 1% (in agarose) and applying an electric potential of 100 V for 35 min. As shown in Figure 2B, when the surface of the AuNPs is not completely functionalized with DNA, the bioconjugate migrates at a faster speed, and the bands generated are broader. However, for a given AuNPs: Agg1 and AuNPs: Agg2 molar ratio, it is observed that the migration speed of the bioconjugates formed remains constant and the bands are narrow, which indicates the formation of homogeneous bioconjugates with a surface of the bioconjugated nanoparticles with DNA strands. The electrophoresis gels shown in Figure 2B indicate that the adequate molar ratios of AuNPs: Agg1 and AuNPs: Agg4 required for an efficient bioconjugation of gold nanoparticles are 1:100 and 1:150, respectively. Thus, the molar ratios were selected for further experiments.

In the development of the sensor presented here, 15 nm nanoparticles have been used for various reasons. Firstly, there is a trade-off between the size of the nanoparticles and the length of the DNA chain used for bioconjugation. If the nanoparticles are too small and the DNA chain is long, aggregation after linker hybridization with the bioconjugated gold nanoparticles becomes difficult because a minimum distance is needed for the nanoparticles to aggregate. Conversely, if the nanoparticles are too large and the DNA chain is short, the nanoparticles will be close enough to aggregate, hindering the recognition of sesame DNA during MNAzyme amplification. Furthermore, using larger nanoparticles requires more DNA sequences for biofunctionalization. For this reason, in the presented work, a 15 nm value for gold nanoparticles was used [18,19].

### 3.3. Optimization of MNAzyme Amplification

In a MNAzyme assay, several parameters can be optimized to achieve the best analytical performance in terms of sensitivity and reproducibility. Some of these key parameters were here optimized, including the magnesium ions’ concentration, which can enhance the efficient cleavage by the MNAzymes. Also, the DNA linker concentration used in the assay can impact its performance as it ensures efficient hybridization and stability of the MNAzyme complex. Therefore, here, the concentration of MgCl_2_ and the linker used in the assay has been evaluated to ensure the aggregation of AuNPs. For this purpose, different molar concentrations of the linker and MgCl_2_ were studied.

**Figure 3:**
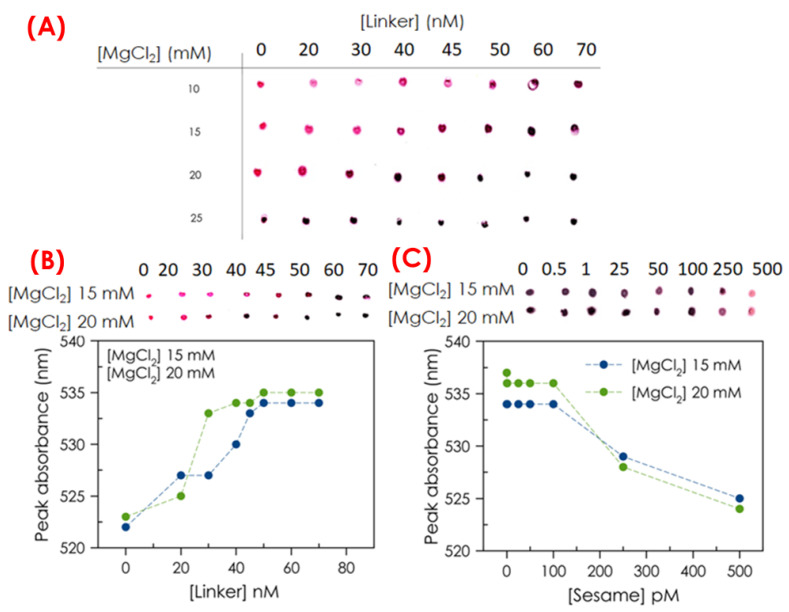
(**A**) Effect of MgCl_2_ and linker concentration on aggregation of AuNPs. Images of AuNPs for optimization of linker and (**B**) MgCl_2_ concentrations using TLC plates. Optimization of (**C**) MgCl_2_ concentration.

The MgCl_2_ concentrations ranging from 10 mM to 25 mM and from 0 nM to 70 nM were prepared for the linker, see Figure 3A,B. The degree of the displacement of the plasmon peak wavelength was the parameter used to select the best experimental conditions for signal amplification. The optimum concentration of MgCl_2_ was found to be between 15 to 20 mM because no aggregation of AuNPs is observed in the absence of a linker. In addition, at this MgCl_2_ concentration, the catalytic efficiency of MNAzymes is guaranteed when the aggregation of AuNPs is observed. A range of concentrations between 50 and 70 nM was tested to optimize linker concentration. As shown in Figure 3A,B, by using linker concentrations higher than 50 nm, AuNP aggregation occurs because the color changes from red (dispersed AuNPs) to blue color (aggregated AuNPs), and the absorption wavelength is shifted to 535 nm. A linker concentration of 50 nM was selected as optimum since it corresponds to the minimum concentration at which there is a higher aggregation of AuNPs.

On the other hand, the concentration of MgCl_2_ (15 and 20 mM) employed was also optimized, fixing the linker concentration of 50 nM in the presence of sesame DNA at concentrations of 0.5, 1, 25, 50, 100, and 500 pM, see Figure 3C. The color change and the displacement of the surface plasmon resonance (SPR) wavelength were established from 100 to 500 pM of sesame DNA in both conditions. According to our results, we decided to use an optimum concentration of MgCl_2_ of 15 mM because it is the lowest concentration in which no aggregation of AuNPs is observed in the presence of the target. Reaction times were also investigated. Longer incubation times may improve sensitivity, but excessively long times were found for non-specific cleavage. The optimum time in the assay was found to be 60 min.

**Figure 4:**
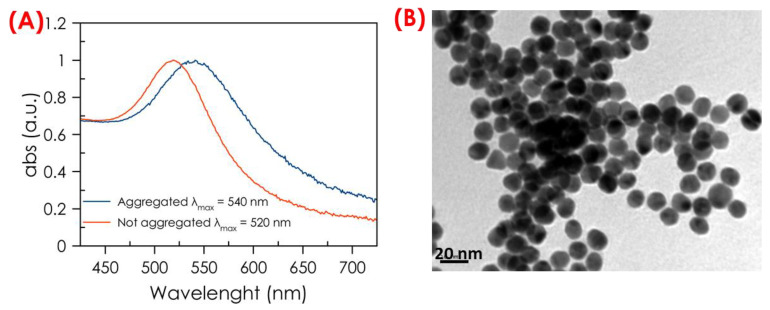
(**A**) UV-Vis absorption spectra of the colorimetric sensing system in the absence (blue line) and presence of sesame DNA (red line). (**B**) TEM images of aggregated AuNPs-DNA without a target.

Furthermore, the absorption wavelengths of the AuNPs were studied using a UV–Vis spectrophotometer. Figure 4A shows the UV–Vis absorbance spectra of non-aggregated (red color) AuNPs and when the NPs are aggregated (blue color). As observed, the wavelength corresponding to the absolute maximum absorbance in the spectrum curve, the plasmon peak λ max, shifts towards higher values as aggregation occurs. In the developed assay strategy, the AuNPs are aggregated when the sample does not contain the sesame DNA target. However, when sesame DNA is present in the sample, the AuNPs are dispersed into the solution. So, the 540 nm peak (aggregated AuNPs) blue-shifts gradually when the concentration of sesame DNA increases. Figure 4B shows a TEM picture of the assay mixture containing the DNA-surface-modified AuNPs after adding a sample without sesame DNA.

### 3.4. Analytical Characterization

**Figure 5:**
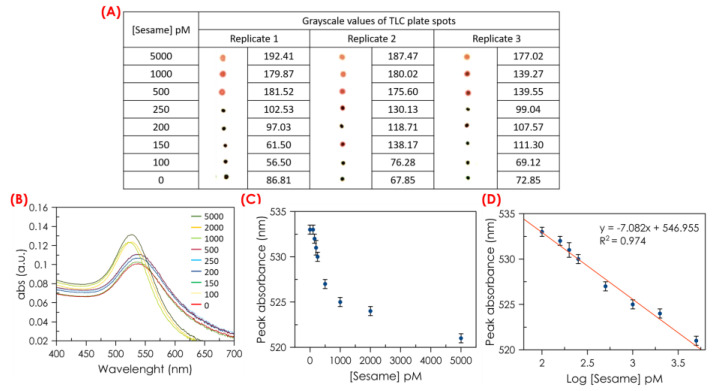
The results of the analytical characterization. (**A**) Images of the colorimetric readout on a TLC plate for each concentration of sesame DNA, and digitalized grayscale values for each spot in the TLC plate. The sesame DNA concentrations were evaluated in triplicate, with columns representing the grayscale values for each spot in the TLC plate. (**B**) Absorbance spectrum peaks for the sesame standard, (**C**) the sensitivity of detecting sesame DNA from 0 pM to 5000 pM, and (**D**) a linear calibration plot for sesame DNA.

Under the optimal conditions, the detection of the linear dynamic range and sensitivity of the colorimetric method for sesame DNA detection were evaluated. The response of the method for sesame DNA standard solutions, in a range of sesame concentration from 0 to 5000 pM, was studied. As shown in Figure 5A, the color of the spots in the TLC indicates the presence (red) and absence (purple or black) of the sesame DNA. The images were adjusted for contrast and brightness. Also, the digitalization of the TLC plate was carried out to obtain the grayscale values for each sample. The grayscale values were calculated using software and were summarized in Figure 5A. Color analysis utilizing image processing software (Ilastik) facilitates the conversion of color into numerical data, enabling a straightforward classification of the samples in the presence of sesame. Meanwhile, in the UV-vis absorption spectra shown in Figure 5B, the absorbance at 541 nm gradually displaced to 520 nm with the increasing sesame DNA concentration. The spectroscopic data showed a shift from 533 nm to 520 nm for sesame DNA concentrations between 100 and 5000 pM. Both spectroscopic measurements and spots in TLC were carried out in triplicate. It must be noted that the presence of sesame DNA even at a concentration above 100 pM does not cause aggregation of the gold nanoparticles, see Figure 5C,D. We observed through color analysis that grayscale values increased with the concentration of sesame DNA. This correlation aligns with expectations in color analysis, considering that in grayscale, dark colors are represented by low-intensity values, thus high-intensity values represent light colors. Typically, image software assigns grayscale values ranging from 0 to 255, with 0 representing black and 255 representing white. All shades of gray therefore fall within this range. This difference in grayscale allows us to distinguish small color changes in samples that would be indistinguishable to the human eye.

Although it may seem at first glance that the absorbance maximum also decreases in samples with low concentrations of sesame DNA, the effect of the wavelength shift is more significant. This shift enables us to differentiate between samples containing sesame DNA and those without. This can be seen in Figure 5C,D. Thus, the detection limit was calculated to be 140 pM. This LOD was determined by considering the mean value minus three standard deviations for the blank sample, which was 531.7 nM. Using the equation included in Figure 5D, the corresponding value of log [sesame] was found to be 2.15, yielding a sesame concentration of approximately 140 pM. However, Figure 5C,D show that only contents around or higher than 150 pM can be quantified. The high sensitivity of the proposed method was derived from the amplification of AuNPs-DNA induced by sesame DNA.

The performance data for the colorimetric detection of sesame allergen using DNA−functionalized gold nanoparticles are presented in Table 2, alongside data from independent studies [3,12,37,38]. These data suggest that integrating biosensor chemistry with color analysis software offers a versatile and powerful platform for the sensitive, on-site, real-time detection of sesame DNA in different samples. Particularly, for allergen detection, highly sensitive detection systems are essential. The effect of allergens on the body depends not only on their concentration. It also depends on the individual’s tolerance to exposure. Therefore, it is necessary to determine the presence of the allergen quickly and with high sensitivity.

For allergen detection, high-detection systems are essential. The effect of allergens on the body depends not only on their concentration. It also depends on the individual’s tolerance to exposure. Therefore, it is necessary to determine the presence of the allergen quickly and with high sensitivity. Consequently, the proposed biosensor here meets these requirements for sesame DNA detection achieving sensitivity levels as low as 140 pM, which is indeed significant.

### 3.5. Sesame DNA Detection in Food Samples

**Figure 6:**
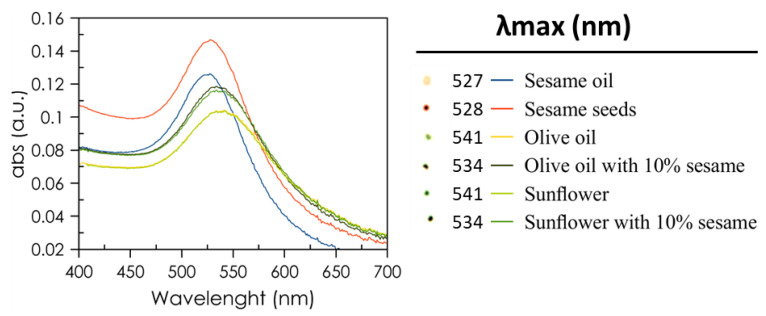
Real sample analysis of sesame DNA in different samples.

The developed procedure was tested to detect sesame DNA in different food samples including sesame oil, sesame seeds, olive oil, and sunflower oil. For olive and sunflower oils, 10% sesame oil was added, simulating olive oil adulteration. As can be seen in Figure 6, the results are skewed towards aggregation when compared with the DNA standards, but there is a clear difference in maximum absorbance wavelength between the sesame-containing samples (~527 nm) and the ones without sesame (541 nm). To verify the potential of the developed method for the quantitative determination of sesame DNA in real food samples, sesame oils and oil samples spiked with sesame DNA were analyzed. The DNA extraction protocol was carried out as described in Section 2.6. Extracting DNA from Food Samples. Once the sesame DNA was extracted, the detection assay was carried out using experimental conditions. Assuming a 70% yield of the DNA extraction [34], the concentration at which a change in color is undoubtedly observed is 78 pM, which agrees with the LOD obtained from this method. Hence, it can be considered that this method can be applied to the analysis of sesame DNA in real samples. Also, the spots in the TLC presented different colors for sesame-containing samples and those without sesame which can be observed with the naked eye.

**Figure 7:**
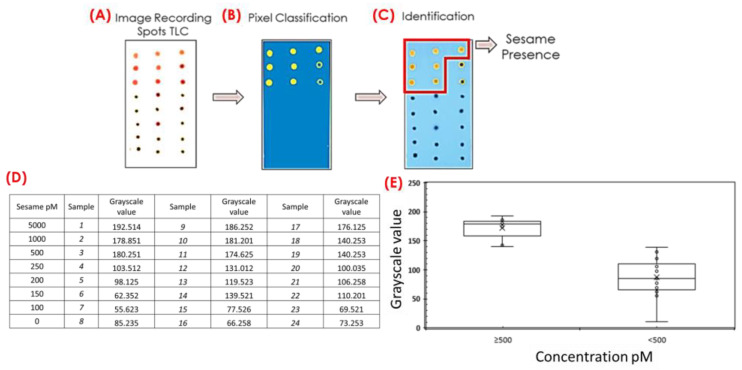
Workflow used for TLC image analysis by Ilastik used to discriminate between aggregated and dispersed AuNPs. (**A**) The raw image of AuNPs after sample addition. (**B**) In the pixel classification step, individual objects are first identified (random color assigned to each particle), and they are classified as aggregated and not aggregated. (**C**) The pixel classification step shows a probability distribution that belongs either to the front (yellow) or the background (blue). (**D**) A table of the colorimetric readout on a TLC plate for all 24 samples of sesame DNA concentration, and digitalized grayscale values. The order of samples in the table (row/column disposition) corresponds to their position in TLC seen in (**A**). (**E**) Box plots of grayscale values for the spots on the TLC plate shown in classification, comparing samples containing different levels of sesame DNA (≥500 or <500 pM).

An interesting alternative to ensure an unbiased visual color-change detection and at the same time to improve the sensitivity and reliability of the assay is to combine MNAzyme amplification and color processing procedures. The use of processing software to discriminate between the aggregation of gold nanoparticles (blue-purple) and non-aggregation (red) was evaluated for the development of a simple, precise, easy-to-handle, and portable analytical method. For such purposes, 3 µL of the assay solution was deposited onto a TLC plate to facilitate the visual differentiation between aggregated and non-aggregated AuNPs. Then, images of TLC captured with a digital camera were used for pattern recognition analysis based on their color values, see Figure 7A.

Color values were calculated using Ilastik, an open-source image analysis software that converts, through segmentation and classification, the color of an image into grayscale values. The general process is based on the loading of an image in jpg or png formats into the program. The software accesses the color values of each pixel, and the image is then automatically segmented and classified by pixel. Different regions of the image are identified and labeled. During this process, the software analyzes the characteristics of each pixel and classifies them based on color intensity (referred to as grayscale). The software then assigns grayscale values to each pixel once the image has been segmented and classified. For the analysis of the spots in the TLC plate, a circular area of the TLC point was selected to reduce noise in the data caused by edge effects and shadows, Figure 7B. The “Pixel Classification” application allows us to use different parameters, including color, and classify each point in the image as not added (presence of sesame) or added (no presence of sesame), which is useful for semiqualitative analysis. The software identifies the red and blue points on the TLC plate. Then, it highlights the pixels (Figure 7C) in yellow for non-aggregated AuNPs and marks the pixels in blue for aggregated AuNPs. These grayscale values are taken directly from the original image and are used to visually represent the different segmented regions. The software supports the exportation of the color analysis results in various formats for further interpretation.

A comparison of the images before and after Ilastik color analysis is shown in Figure 7C, showing marked differences between samples. The software classifies samples based on their numerical conversion (Figure 7B,D). Visually, samples deposited on the TLC plate can be distinguished as either having the presence of sesame DNA (red dots) or no presence of sesame DNA (blue dots); however, some samples are not easily identifiable through visual inspection alone. Color analysis utilizing image processing software facilitates the conversion of color into numerical data (see Figure 7D), enabling a straightforward classification of the samples in the presence of sesame (see Figure 7C). This represents a significant utility of the image processing software employed in this study, enhancing assay sensitivity. A total of 24 samples with concentrations between 100 and 5000 pM of sesame DNA and without sesame were analyzed using the proposed procedure, as can be seen in Figure 7D. Color analysis using Ilastik software enabled us to classify the samples into two groups: samples with a sesame DNA concentration ≥500 pM and samples with a concentration <500 pM, see Figure 7E.

## 4. Conclusions

This work reports a new colorimetric bioanalytical method for simple, efficient, specific, and highly sensitive detection of trace levels of sesame DNA in food samples. It has been demonstrated that the combination of DNA-surface-modified AuNPs with a signal amplification strategy based on MNAzymes allows for the detection of the DNA target with a high selectivity providing very low detection limits using spectrophotometric measurements (140 pM). Also, spotting onto a TLC plate of a few microliters of the assay solution, in combination with the use of open-source software for color signal processing, greatly facilitates the visualization of the color differences, minimizing the effects derived from differences in the perception of the color due to ambient light. The assay was successfully tested for the determination of sesame in contaminated olive and sunflower oils and in sesame seeds.

In brief, this methodology can be used in a wide range of laboratories for food safety control because it is easy to use, specific, and highly sensitive. Considering that the analytical signal is only a color change, there is no need for trained personnel to perform the analysis. Moreover, the developed assay could be applied to the detection of some other different genetic material characteristic of a wide variety of toxins, adulterants, or contaminants eventually present in food samples, due to the inherent properties of MNAzymes. The future of the groundbreaking colorimetric bioanalytical method for detecting sesame DNA in food samples is highly promising. This versatile method can be adapted to identify various genetic materials from different contaminants, enhancing food safety across diverse products. This work also paves the way for the use of digital cameras and open-source color signal processing software, enabling rapid and accurate analysis. This makes the method accessible in resource-limited settings. Future advancements may integrate the assay with portable devices for on-site testing, automate high-throughput screening, and improve sensitivity and specificity. Additionally, potential applications in food and health monitoring highlight its broad utility and potential impact on public health and environmental protection.

## Figures and Tables

**Table 1:** DNA sequences of sesame target and MNAzymes.

Designation	Sequence (5′→3′)	Tm °C
Linker *	AGC ATG GTC GAT AGG TAA GGT TTC CTC rGrUC CCT GGG CAT AAA CGA CTC TAG CGC	70.1
Strand 1 (Agg1)	HS-AAA AAA AAA ACC TAT CGA CCA TGC T	52.69
Strand 2 (Agg4)	GCG CTA GAG TCG TTT AAA AAA AAA A-SH	51.74
AF_Ses1	GGG CTA TCT TTC AAG CGT GCG AAT GAA CCC TTC AGT GGT ACG GAG TC	63.6
MNAzymes R	GAC TCC GTA CCA CTG AAG GGT TCA ACA ACG AGA GGA AAC CTT	66.23
MNAzymes L	TGC CCA GGG AGG CTA GCT TTC GCA CGC TTG AAA GAT AGC CC	42.86
AF_Ses2	TCT TTC AAG CGT GCG AAT GAA CCC TTC AGT GGT ACG GAG TC	69.60
MNAzymes R	GAC TCC GTA CCA CTG AAG GGA CAA CGA GAG GAA ACC TT	68.68
MNAzymes L	TGC CCA GGG AGG CTA GCT TTC ATT CGC ACG CTT GAA AGA	71.80
AF_Ses3	GGG CTA TCT TTC AAG CGT GCG AAT GAA CCC TTC AGT GGT AC	48.9
MNAzymes R	GTA CCA CTC AAG GGT TCA TTA CAA CGA GAG GAA ACC TT	64.51
MNAzymes L	TGC CCA GGG AGG CTA GCT CGC ACG CTT GAA AGA TAG CCC	75.00
AF_Ses4	TCC TTT GAA TTT TTG GGC TAT CTT TCA AGC GTG CGA ATG AAC CCT	67.31
MNAzymes R	TCC TTT GAA TTT TTG GGC TAT CTT TAC AAC GAG AGG AAA CCT T	63.30
MNAzymes L	TGC CCA GGG AGG CTA GCT CAA GCG TGC GAA T GA ACC CT	75.13

Tm: melting temperature; AF_Ses1, AF_Ses2, AF_Ses3, and AF_Ses4: sesame targets assayed; *: r in sequence indicates the cleavage site in the linker strand.

**Table 2:** Comparison between some selected biosensors for sesame determination.

Material	Analytical Method	Sensor Principle	Lineal Range (pM)	LOD (pM)	Reference
QDs-OVA, AuNPs-Ab	Fluorescence	Immuno-chromatographic test strips	51,200	3200	[3]
MPMs-SA, QDs-Ab	Fluorescence	Immunoassay	3200–25,600	154	[12]
HRP-goat anti-mouse antibody	Colorimetric	Double antibody sandwich ELISA	–	20,000	[37]
Aptamer-AuNPs	Colorimetric	Aptamer–target binding	0–10,000	500	[38]
DNA-AuNPs	Colorimetric	MNAzyme amplification and free color image analysis	100–5000	140	This work

## Data Availability

Data are contained within the article.
